# Pure Cerebellar Ataxia with Homozygous Mutations in the *PNPLA6* Gene

**DOI:** 10.1007/s12311-016-0769-x

**Published:** 2016-03-19

**Authors:** Sarah Wiethoff, Conceição Bettencourt, Reema Paudel, Prochi Madon, Yo-Tsen Liu, Joshua Hersheson, Noshir Wadia, Joy Desai, Henry Houlden

**Affiliations:** 1 Department of Molecular Neuroscience, UCL Institute of Neurology, London, UK; 2 Center for Neurology and Hertie Institute for Clinical Brain Research, Eberhard-Karls-University, Tübingen, Germany; 3 National Hospital for Neurology and Neurosurgery, UCL Institute of Neurology, Queen Square, London, WC1N 3BG UK; 4 Department of Clinical and Experimental Epilepsy, UCL Institute of Neurology, London, UK; 5 Department of Assisted Reproduction and Genetics, Jaslok Hospital and Research Centre, Mumbai, 400026 India; 6 Section of Epilepsy, Department of Neurology, Neurological Institute, Taipei Veterans General Hospital, Taipei, Taiwan; 7 National Yang-Ming University School of Medicine, Taipei, Taiwan; 8 Department of Neurology, Jaslok Hospital and Research Centre, Mumbai, India

**Keywords:** Cerebellar ataxia, Gene, Mutations, *PNPLA6*

## Abstract

Autosomal-recessive cerebellar ataxias (ARCA) are clinically and genetically heterogeneous conditions primarily affecting the cerebellum. Mutations in the *PNPLA6* gene have been identified as the cause of hereditary spastic paraplegia and complex forms of ataxia associated with retinal and endocrine manifestations in a field where the genotype-phenotype correlations are rapidly expanding. We identified two cousins from a consanguineous family belonging to a large Zoroastrian (Parsi) family residing in Mumbai, India, who presented with pure cerebellar ataxia without chorioretinal dystrophy or hypogonadotropic hypogonadism. We used a combined approach of clinical characterisation, homozygosity mapping, whole-exome and Sanger sequencing to identify the genetic defect in this family. The phenotype in the family was pure cerebellar ataxia. Homozygosity mapping revealed one large region of shared homozygosity at chromosome 19p13 between affected individuals. Within this region, whole-exome sequencing of the index case identified two novel homozygous missense variants in the *PNPLA6* gene at c.3847G>A (p.V1283M) and c.3929A>T (p.D1310V) in exon 32. Both segregated perfectly with the disease in this large family, with only the two affected cousins being homozygous. We identified for the first time *PNPLA6* mutations associated with pure cerebellar ataxia in a large autosomal-recessive Parsi kindred. Previous mutations in this gene have been associated with a more complex phenotype but the results here suggest an extension of the associated disease spectrum.

**Electronic supplementary material:**

The online version of this article (doi:10.1007/s12311-016-0769-x) contains supplementary material, which is available to authorized users.

## Introduction

Autosomal-recessive cerebellar ataxia (ARCA) comprises a heterogeneous group of neurodegenerative conditions inherited in autosomal-recessive fashion with primary affection of the cerebellum. There are variable associated neurological signs such as spasticity, seizures, optic atrophy, neuropathy and cognitive impairment with an ever-growing number of causative genes identified [1, 2]. With the advent of whole-exome and whole-genome sequencing (WES and WGS) for research and diagnostics, gene discovery has advanced in many ways with new genes and genotype-phenotype correlations [3].

Mutations in the patatin-like phospholipase domain-containing protein 6 (*PNPLA6)* gene were originally described in 2008 as causative for specific forms of complicated hereditary spastic paraplegia (SPG39, MIM#612020) [4]. Recently, using a whole-exome sequencing approach, *PNPLA6* mutations were additionally identified as the most frequent unifying genetic cause of two distinct clinical syndromes with previously elusive genetic origin: Boucher-Neuhauser (BNHS, MIM #215470) and Gordon Holmes syndromes (GDHS, MIM #212840). Subsequently, this gene was associated with a broad neurodegenerative spectrum, frequently including ataxia, motor neuron disease, chorioretinal dystrophy and hypogonadism [5, 6], and recently, *PNPLA6* was independently identified as the genetic cause in several families with Laurence-Moon syndrome (LNMS, MIM #245800), Oliver-McFarlane syndrome (OMCS, MIM #275400) and Leber congenital amaurosis (LCA1, MIM #204000) [7, 8].

Here, we report a large consanguineous Indian Parsi family with pure cerebellar ataxia in two affected cousins. Using homozygosity mapping and whole-exome sequencing, two novel homozygous missense changes in the *PNPLA6* gene were identified. To our knowledge, this is the first report associating *PNPLA6* mutations with pure cerebellar ataxia.

## Patients and Methods

### Patients

**Fig. 1:**
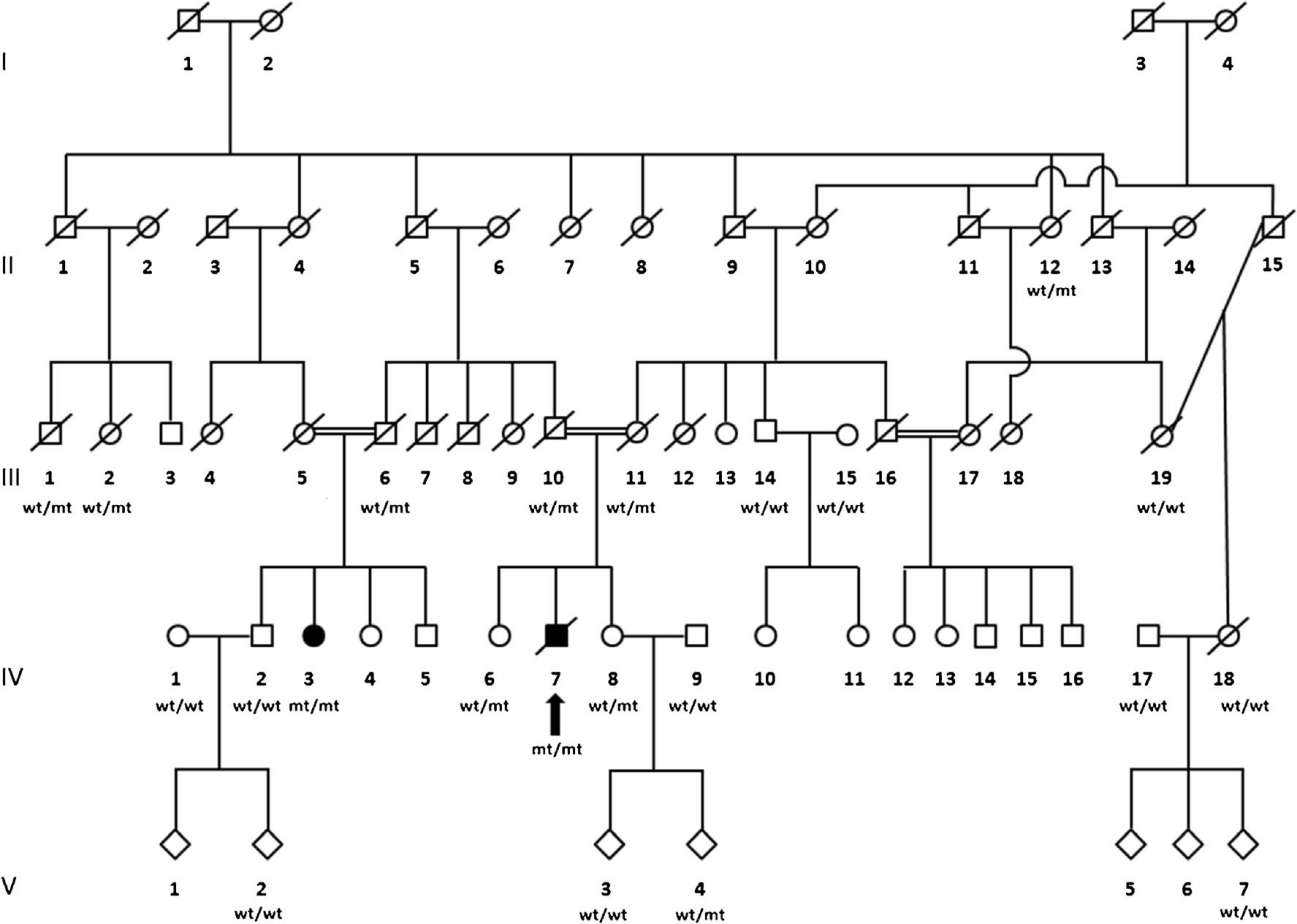
Family tree with segregation status of homozygous changes at positions c.3847G>A, p.V1283M and c.3929A>T, p.D1310V (*wt* wildtype, *mt* mutant, the *arrow* denotes the index case)

The patients were from a large, multigenerational consanguineous family of Zorastrians (Parsis) of Indian origin with two affected cousins, both born to consanguineous first-cousin marriages (Fig. 1). The Zoroastrians are a tiny and closed community who are followers of the pre Christian prophet Zoroaster/Zarathustra.

The index patient was first seen 55 years ago at the age of 16 (NW, Mumbai), diagnosed as having spinocerebellar ataxia (SCA) and underwent periodic clinical follow-up. At the age of 57, he was seen together with his affected cousin at the Department of Assisted Reproduction and Genetics, Jaslok Hospital where thorough neurological and general medical examination (JD), pedigree analysis, karyotyping and chromosome breakage study were carried out, genetic counselling given and further testing coordinated. SCAs 1, 2, 3, 6, 7, 11, and 15, DRPLA, Friedreich ataxia, Fragile-X and Huntington disease were excluded and a genetic diagnosis could not be reached.

### Genetic Analyses

With the approval of the joint ethics committee of UCL Institute of Neurology and the National Hospital for Neurology and Neurosurgery, London, UK (UCLH: 04/N034), DNA and RNA were extracted from blood samples of both cousins and 20 unaffected relatives who had given their consent using standardized protocols. To determine the genetic cause of the disease, we performed homozygosity mapping in the two affected cousins and two unaffected family members by using HumanOmniExpress BeadChip Kit (Illumina) and analysing the data with HomozygosityMapper (http://www.homozygositymapper.org/). Subsequently, WES was carried out in the index case using NimbleGen SeqCap Target Enrichment EZ-system (Roche Sequencing) and Illumina 76 bp paired end sequencing on a GAII platform as part of a commercial service. Provided annotated variant files were analysed for homozygous non-synonymous variants in the homozygous stretches previously determined and other known genetic causes of cerebellar ataxia outside these regions and detectable via exome sequencing were confirmatory excluded. Sanger sequencing was performed to confirm variants, test segregation, and screen an additional cohort of 40 British pure cerebellar ataxia patients (primers available upon request). To rule out possible effects of mutations on splicing, cDNA was created from RNA and subsequently Sanger sequenced.

## Results

### Clinical Description

**Fig. 2:**
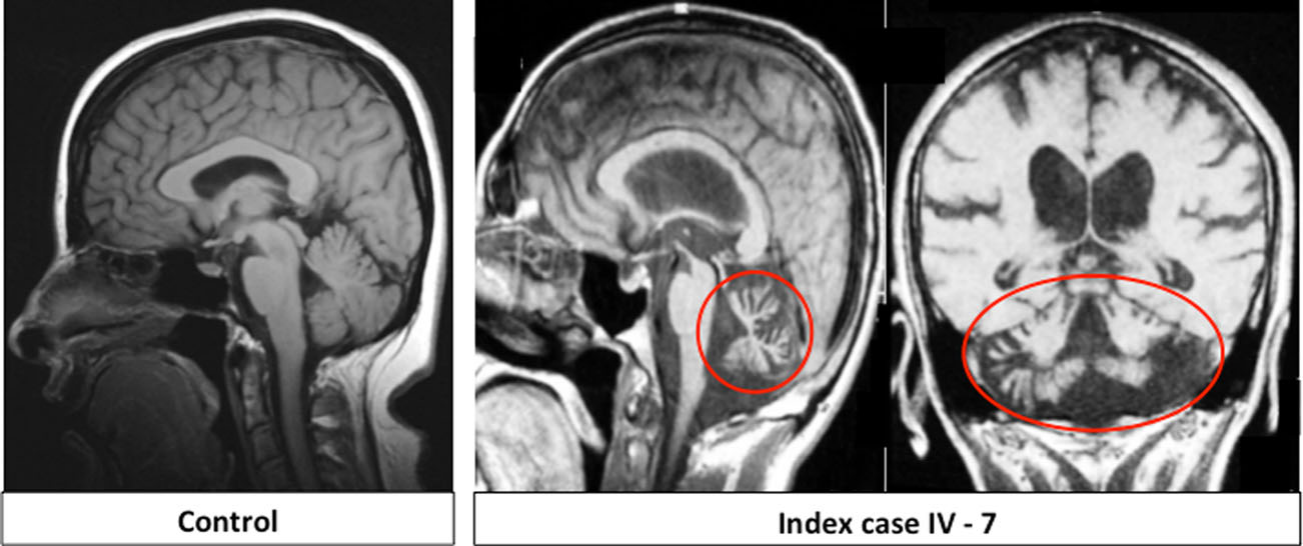
T1-weighted sagittal MRI of unaffected control proband (*left*) and sagittal and coronal MRI of index case at age 60 depicting severe cerebellar atrophy circled in *red* and some additional degree of midbrain atrophy

The index patient (Fig. 1: IV–7) developed slowly progressive cerebellar symptoms with balance and coordination problems primarily affecting gait and manual movements at the age of 12. He rarely cried as an infant, had nystagmus and poor handwriting and was a double graduate, though needing a writer for exams. The index case’s condition was significantly exacerbated by a stroke due to thrombosis in the left transverse sinus at age 60. In this context, an MRI was acquired that showed severe cerebellar and some degree of midbrain atrophy (Fig. 2), and polycythemia vera was diagnosed and subsequently controlled with medication and phlebotomy. It was only after the stroke that vision was impaired due to the ischaemic event and subsequent falls led to parenchymal and subdural haematomas causing aphasia, disorientation, aggression, a very strong grip with stereotypy of hand and neck with the head tilting backwards. He died one year ago at the age of 70. Regarding the movement disorder, the paternal cousin of the index patient (Fig. 1: IV−3, currently at age 68) developed similar symptoms around puberty with pure cerebellar ataxia leading to frequent falls and leg fractures, for which she is now wheelchair bound. She has dysmetric slow saccades and besides poor handwriting, she does not have any further complicating symptoms being mentally alert, though speech is slow. In both affected individuals, ataxic gait and brisk lower limb reflexes suggested early onset cerebellar ataxia with retained reflexes (EOCARR). Nerve conduction studies were within normal limits. EMG recordings were suggestive of bilateral S1 segment lesion at the proximal level for both patients and additional left L5 segment lesion at proximal level for the affected cousin (IV−3). There was no hypogonadotropic hypogonadism or chorioretinal dystrophy in both of them. Clinical data was available from 22 individuals of the family (Fig. 1), whereof only the two reported cousins were found symptomatic.

### Genetic Analyses

**Fig. 3:**
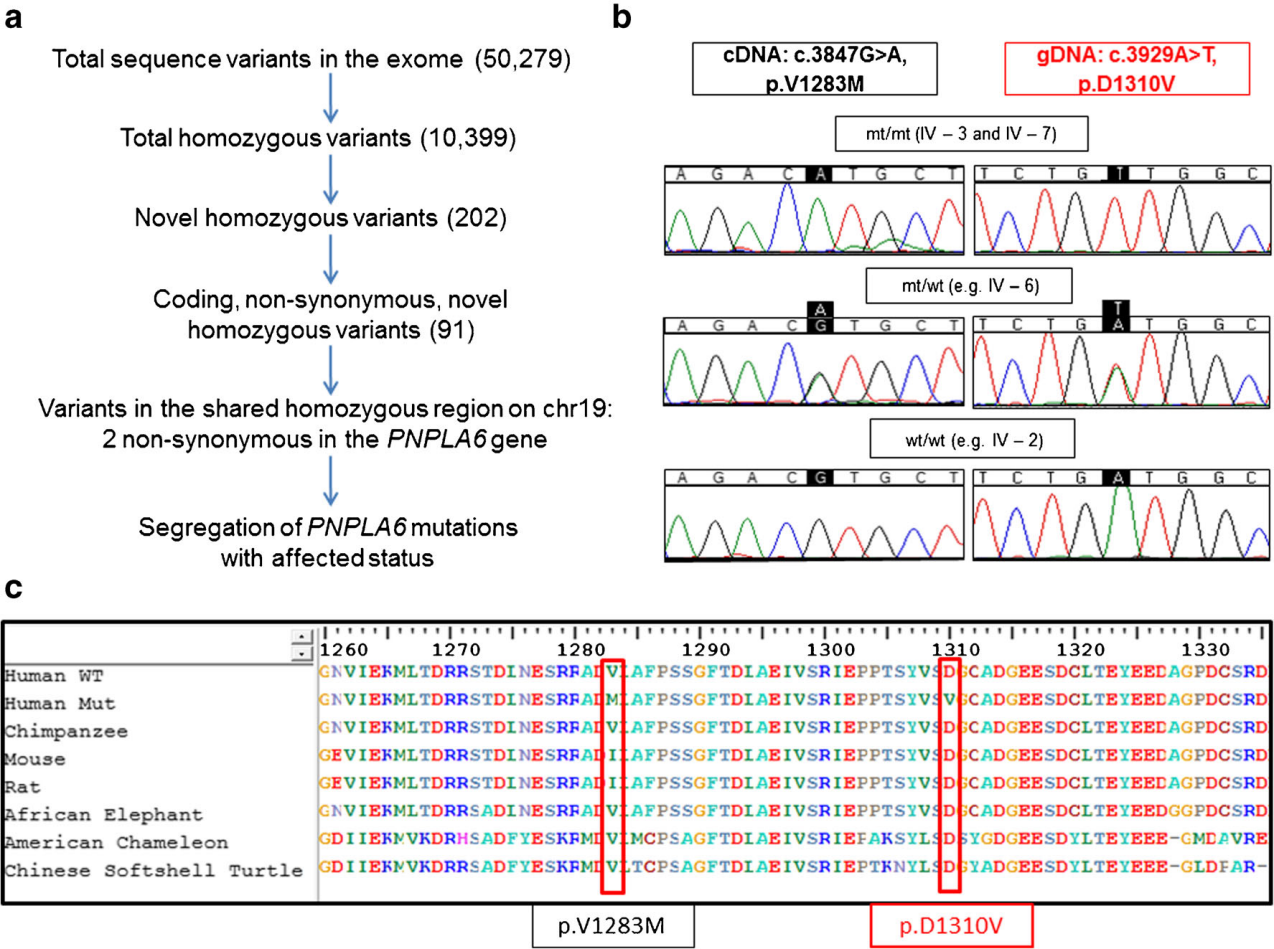
**a** Filtering strategy for variants in the exome of the index case. **b** Sanger sequencing of affected index case with homozygous changes at positions c.3847G>A, cDNA and c.3929A>T, gDNA (*top-panels*), heterozygous unaffected (*mid-panel*), and wildtype unaffected relatives (*lower panel*). No effects on splicing were observed for the c.3847G>A mutation. **c** Multiple sequence alignment showing conservation across species at the affected amino acid residues 1283 and 1310 (*WT* wildtype, *Mut* mutant)

Homozygosity mapping revealed only one large region of shared homozygosity by the two affected cousins on chromosome 19 (Supplementary Figure 1), spanning over 4 Mb (Chr19: 3630740–7759053). This region comprises 132 genes, from which two (*ATCAY*, *PNPLA6*) were particularly interesting candidates given the phenotype of our patients. WES revealed one synonymous change in *SAFB2* (unlikely to be related with the disease) and two homozygous missense changes in *PNPLA6* at c.3847G>A (p.V1283M) and c.3929A>T (p.D1310V) in exon 32, transcript ENST00000414982 (see Fig. 3a for filtering strategy). The *PNPLA6* variants were confirmed by Sanger sequencing (Fig. 3b) and segregated perfectly with the disease in all 20 unaffected and two affected individuals tested (Figs. 1 and 3b): Nine heterozygous carriers were detected in four generations, and only the two affected cousins were homozygous. All exons of *ATCAY* and *PNPLA6* were also screened by Sanger sequencing and ruled out the presence of additional variants that could have been missed by WES. Screening of 40 British patients with pure cerebellar phenotype revealed no further mutations in *PNPLA6*. No additional mutations in known autosomal recessive cerebellar ataxia genes were detectable on the exome.

The variants found in our consanguineous family (p.V1283M and p.D1310V, see Table 1 for summary) are not present in public databases (Exome Sequencing Project variant server (EVS), dbSNP, Exome Aggregation Consortium (ExAC), Complete Genomics 69 (cg69) and 1000 Genomes). The *PNPLA6* c.3929A>T, p.D1310V was conserved across species (Table 1 and Fig. 3c), and predicted to be disease causing by three prediction tools (Table 1). Since the variant c.3847G>A, p.V1283M, with more benign prediction scores (Table 1), lies at the first nucleotide of exon 32, we investigated potential effects on splicing (e.g. exon skipping) by Sanger sequencing cDNA from the affected cousins and their carrier and wildtype siblings. No splicing changes were detected around exon 32 (see Fig. 3b).

**Table 1:** Frequency and in silico predictions of homozygous, novel missense *PNPLA6* variants identified in large Parsi-pedigree

Chr	Position (hg19)	Transcript	Variant	dbSNP, 1000 g, ExAC, EVS, cg69	GERP score^a^	SIFT^b^	Mutation taster	PolyPhen2
19	7625900	ENST00000414982	c.3847G>A, p.V1283M	absent	2.79	T	N	B
19	7625982	ENST00000414982	c.3929A>T, p.D1310V	absent	3.81	D	D	P

*GERP* Genomic Evolutionary Rate Profiling, *SIFT* ‘Sorting Tolerant From Intolerant’, *D* deleterious/damaging/disease-causing, *P* possibly damaging, *N* polymorphism, *T* tolerated, *B* benign

^a^Positive conservation scores represent a substitution deficit and indicate that a site may be under evolutionary constraint. Negative scores indicate that a site is probably evolving neutrally. Positive scores scale with the level of constraint, such that the greater the score, the greater the level of evolutionary constraint inferred to be acting on that site

^b^Using the ‘Sorting Tolerant From Intolerant’ algorithm [9], this tool predicts whether an amino acid substitution affects protein function based on the degree of conservation of amino acid residues in sequence alignments derived from closely related sequences

## Discussion

We identified the first *PNPLA6* mutations in a large kindred of Indian Zorastrians (Parsis) affected by a pure autosomal-recessive cerebellar syndrome. This patatin-like phospholipase domain-containing 6 gene on chromosome 19 encodes neuropathy target esterase (NTE), a phospholipase that produces glycerophosphocholine by deacetylation of intracellular phosphatidylcholine. It therefore has important roles in membrane axonal integrity, phospholipid trafficking and phosphatidylcholine-metabolism [10–12]. NTE-activity can be impaired by exposure to neurotoxic organophosphorous (OP) compounds which can result in OP compound-induced delayed neuropathy (OPIDN) and further neurological symptoms [13]. Clinical features are in part similar to the widespread symptoms we observe upon genetic impairment of NTE due to mutations in *PNPLA6* [5, 7, 8].

Recently, reports of biallelic mutations in the *PNPLA6* gene originally causative for SPG39 [4] have extended the phenotypic spectrum, frequency and geographic occurrence [14], e.g. compound heterozygous mutations have been reported in a sporadic BNS-patient with late-onset gait ataxia and mild retinal changes [15], the first two non-Caucasian *PNPLA6* cases of Japanese origin have been published [16], and *PNPLA6* was identified as the genetic cause in several families with Laurence-Moon syndrome (LNMS, MIM #245800), childhood blindness, Oliver-McFarlane syndrome (OMCS, MIM #275400) and Leber congenital amaurosis (LCA1, MIM #204000) [7, 8]. Here, we present two novel homozygous variants associated for the first time with a pure cerebellar phenotype in a large kindred of Indian descent. The more conserved and predicted deleterious variant (c.3929A>T, p.D1310V) lies between the previously published pathogenic mutation c.3365C>T, p.P1122L, located in the catalytically active phospholipid-esterase-domain and shown to cause BNS, and the farthest downstream pathogenic mutation towards the C-terminal site, c.4048C>G, p.R1362G, causing GHS [5]. It might therefore not have a direct diminishing effect within the catalytic center, but an indirect steric inhibition of the protein’s original conformation is possible and its close proximity to the recently identified mutational cluster within the C-terminal phospholipid esterase domain might supports its possibly pathogenic role [5]. The variant c.3847G>A, p.V1283M has non-deleterious prediction scores and is likely to be a tolerated missense variant in linkage disequilibrium. An additional impact of this predicted benign variant towards the phenotype cannot be ruled out though and until further proof of functional pathogenicity becomes available the final interpretation of both novel variants remains unclear.

## Conclusion

According to all published reports to date, both variants we found in *PNPLA6* are novel and represent the first report of *PNPLA6* variants in individuals of Indian descent. This report suggests a possible extension of the clinical spectrum of *PNPLA6* associated diseases to pure cerebellar ataxia and argues for *PNPLA6*-testing to be considered in cases of early-onset cerebellar ataxia despite the absence of chorioretinal dystrophy or hypogonadotropic hypogonadism that are regularly associated with mutations in this gene [5, 6, 17].

## Web Resources

The URLs for data presented herein are as follows:

Online Mendelian Inheritance in Man (OMIM), http://www.omim.org/

Homozygosity Mapper: http://www.homozygositymapper.org/

NHLBI Exome Variant Server EVS: evs.gs.washington.edu

1000 Genomes project: www.1000genomes.org

Complete Genomics cg69 database: www.completegenomics.com/public-data/69-Genomes

dbSNP: www.ncbi.nlm.nih.gov/projects/SNP

Exome Aggregation Consortium database: http://exac.broadinstitute.org/

MutationTaster: http://www.mutationtaster.org/

PolyPhen2: http://genetics.bwh.harvard.edu/pph2

SIFT: http://sift.jcvi.org/

CADD: http://cadd.gs.washington.edu/home

## Electronic Supplementary Material

Below is the link to the electronic supplementary material.

Supplementary Figure 1 Blocks of homozygosity along chromosome 19. The arrow points to the homozygosity stretch shared by the affected cousins only (panel 2 and panel 4 from the top). (GIF 272 kb)

High Resolution (TIF 243 kb)
