# Supplementary figures and images for: Pure Cerebellar Ataxia with Homozygous Mutations in the PNPLA6 Gene
Source: Cerebellum. 2016 Mar 19;16(1):262–7. doi: 10.1007/s12311-016-0769-x (PMC5243903; doi:10.1007/s12311-016-0769-x)

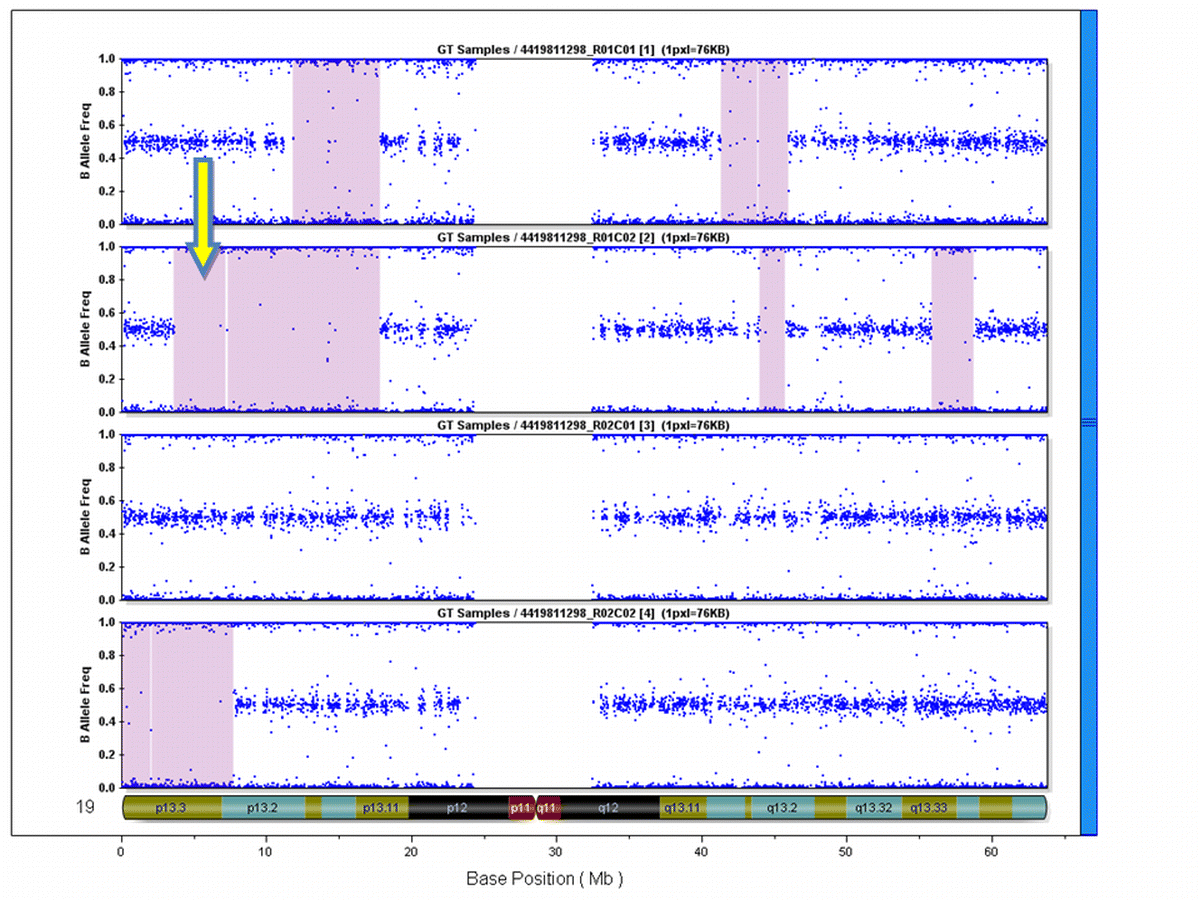

Supplement: Supplementary file 1 — Blocks of homozygosity along chromosome 19. The arrow points to the homozygosity stretch shared by the affected cousins only (panel 2 and panel 4 from the top). (GIF 272 kb) [file 12311_2016_769_Fig4_ESM.gif]

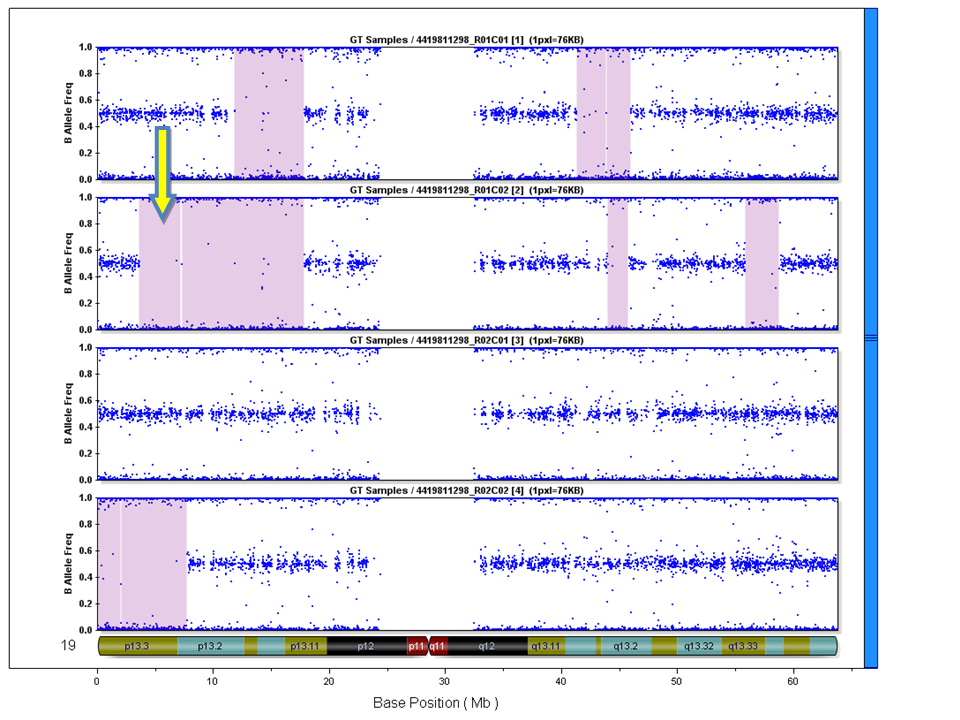

Supplement: Supplementary file 2 — High Resolution (TIF 243 kb) [file 12311_2016_769_MOESM1_ESM.tif]
